# Multiplex proteomics identifies inflammation-related plasma biomarkers for aging and cardio-metabolic disorders

**DOI:** 10.1186/s12014-024-09480-x

**Published:** 2024-04-22

**Authors:** Siting Wu, Yulin Li, Xue Zhao, Fu-Dong Shi, Jingshan Chen

**Affiliations:** 1 Department of Neurology, Tianjin Neurological Institute, Tianjin Medical University General Hospital, Tianjin, 300070 China; 2 Center for Neurological Diseases, China National Clinical Research Center for Neurological Diseases, Beijing Tiantan Hospital, Capital Medical University, Beijing, 100070 China

**Keywords:** Cardio-metabolic disorders, Aging, Multiplex proteomics, Plasma biomarkers, Inflammation

## Abstract

**Background:**

Cardio-metabolic disorders (CMDs) are common in aging people and are pivotal risk factors for cardiovascular diseases (CVDs). Inflammation is involved in the pathogenesis of CVDs and aging, but the underlying inflammatory molecular phenotypes in CMDs and aging are still unknown.

**Method:**

We utilized multiple proteomics to detect 368 inflammatory proteins in the plasma of 30 subjects, including healthy young individuals, healthy elderly individuals, and elderly individuals with CMDs, by Proximity Extension Assay technology (PEA, O-link). Protein-protein interaction (PPI) network and functional modules were constructed to explore hub proteins in differentially expressed proteins (DEPs). The correlation between proteins and clinical traits of CMDs was analyzed and diagnostic value for CMDs of proteins was evaluated by ROC curve analysis.

**Result:**

Our results revealed that there were 161 DEPs (adjusted *p* < 0.05) in normal aging and EGF was the most differentially expressed hub protein in normal aging. Twenty-eight DEPs were found in elderly individuals with CMDs and MMP1 was the most differentially expressed hub protein in CMDs. After the intersection of DEPs in aging and CMDs, there were 10 overlapping proteins: SHMT1, MVK, EGLN1, SLC39A5, NCF2, CXCL6, IRAK4, REG4, PTPN6, and PRDX5. These proteins were significantly correlated with the level of HDL-C, TG, or FPG in plasma. They were verified to have good diagnostic value for CMDs in aging with an AUC > 0.7. Among these, EGLN1, NCF2, REG4, and SLC39A2 were prominently increased both in normal aging and aging with CMDs.

**Conclusion:**

Our results could reveal molecular markers for normal aging and CMDs, which need to be further expanded the sample size and to be further investigated to predict their significance for CVDs.

**Supplementary Information:**

The online version contains supplementary material available at 10.1186/s12014-024-09480-x.

## Introduction

In an aging population, cardiovascular diseases (CVDs) have a high prevalence and will result in 40% of all deaths, ranking as the leading cause [1]. As an independent risk factor for cardiovascular diseases, aging leads to progressive deterioration of the cardiovascular structure and function manifested as cardiac and vascular remodeling, dampened cardiac function, endothelial defects, and loss of vascular compliance [2, 3]. Cardiometabolic disorders (CMDs) are a cluster of risk factors significantly increasing the risk of CVDs, such as hyperlipidemia and hyperglycemia, which are common in elderly individuals [4]. Knowledge of the molecular mechanism common to normal aging and CMDs will assist in providing interventions to prevent or delay the onset of cardiovascular diseases. Identification of the molecular changes provides not only the potentially predicted risk factor for CMDs and even CVDs in normal aging but also provides clues on the molecular mechanism by which aging is involved in the pathogenesis of CMDs.

Increasing evidence supports the idea that aging renders the body in a state of low-grade inflammation with an upregulation of IL-6, TNF-α, and IL-8 [5, 6], which predicts frailty in older adults and is associated with the risk of mortality in healthy elderly individuals [7]. Chronic tissue inflammation, caused by senescent cell accumulation on-site through the secretion of proinflammatory growth factors, cytokines, and chemokines, contributes to the development of some aging-related diseases, including atherosclerosis, diabetes, and cancer [8]. Likewise, inflammation is a key factor in the initiation of CMDs. Because systemic metabolic abnormalities tend to be closely associated with fat accumulation in obesity, inflammatory pathway activation of adipose cells under stress increases proinflammatory cytokines (TNF-α) and chemokines and local infiltration of immune cells, which reduces metabolic flexibility and impairs insulin receptor signaling. This leads to a reduction in the removal of glucose from the bloodstream and increases lipolysis, contributing to hyperglycemia and hypertriglyceridemia [9, 10]. Although some inflammatory proteins were reported to play an important role in both aging and CMDs, how their inflammatory molecular phenotypes and their common molecular changes the common inflammatory molecular alteration is still unknown.

Circulating proteins store rich information regarding an individual’s pathophysiology since they are the final effectors of pathophysiological pathways. There are several advanced technologies applied in plasma proteomics, such as mass spectrometry (MS)-based proteomics, a multiplexed proteomic assay using modified aptamers (SOMAscan), and a proximity extension assay (PEA, O-Link) [11–13]. Of these, PEA technology possesses exceptional readout specificity and sensitivity (sub-pg/mL), enabling high multiplex assays with coverage across a broad dynamic range (∼9 log) while consuming a minimal amount of samples by using unique antibody–oligonucleotide protein binding for quantitative real-time polymerase chain reaction (PCR)-based measurement [14, 15]. It has been used to detect plasma biomarkers for many disease types [16–19].

In our study, 368 inflammatory proteins in the plasma of healthy young (HY) individuals, healthy elderly (HE) individuals, and elderly individuals with CMDs were detected using PEA technology (O-link) to explore comprehensive inflammatory molecular phenotypes in the plasma of aging and CMDs and their common molecular expression, which may provide potential biomarkers for aging and CMDs and clues on the molecular mechanism by which aging is involved in the pathogenesis of CMDs.

## Materials and methods

### Subjects and samples

**Fig. 1:**
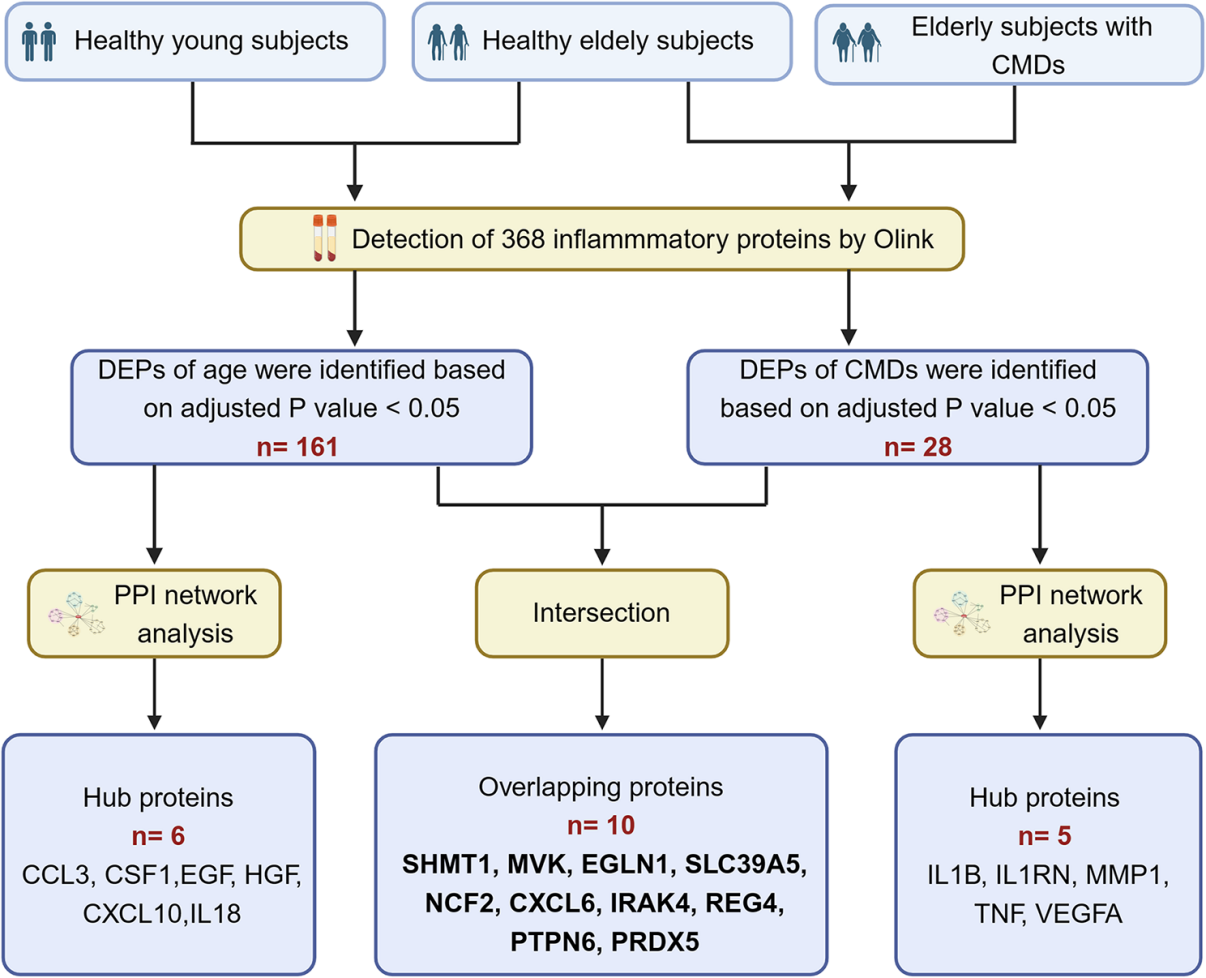
Flow chart of this study. Schematic diagram shows the experiment design. CMD = Cardiometabolic disorders. DEPs = Differentially expressed proteins. PPI network = protein-protein interaction PPI

The schematic diagram shows the experimental design (Fig. 1). Thirty participants were recruited for this study, including 10 HY subjects (aged below 60 years), 10 HE subjects, and 10 elderly subjects with CMDs. CMDs refer to the pathological condition characterized by the aberrant coalescence of various metabolic elements within an individual, significantly increasing the susceptibility to cardiovascular diseases. These metabolic perturbations includes: (1) abdominal adiposity or adipose tissue excess, (2) atherogenic dyslipidemia (elevated triglycerides and reduced HDL-C), and (3) insulin resistance and/or disturbances in glucose tolerance [20]. Elderly subjects were included in CMDs according to the following criteria: (1) triglycerides > 1.7 mmol/L or high-density lipoprotein cholesterol (HDL-C) < 1.04. (2) fasting plasma glucose (FPG)>5.6 mmol/L. (3) waist circumference ≥ 90 cm in men or ≥ 85 cm in women. As evidenced by physical and clinical examinations, the healthy young and elderly subjects were in good health with normal metabolic level. The exclusion criteria were prior myocardial infarction or stroke, morbid obesity, tumor, administration of hypoglycemic drugs, lipid-lowering drugs, and/or immunosuppressive drugs. The plasma sample of subjects was processed following the protocol that was approved by the institutional review board of Tianjin Medical University General Hospital (Tianjin, China). Written informed consent was obtained from all participants.

### Proteomic quantification

Protein concentrations were quantified using multiplex immunoassay, developed by O-link Proteomics (Uppsala, Sweden) and based on PEA technology. This assay converts the measurement of protein concentration to Ct values using qPCR and then reports the protein concentration as normalized protein expression (NPX) on a log2 scale by a normalization procedure. A customized multiplex panel was used to detect 368 inflammatory proteins.

### Functional enrichment analysis

The Database for Annotation, Visualization, and Integrated Discovery (DAVID) function annotation tool (https://david.ncifcrf.gov) was used to perform Gene Ontology (GO) and Kyoto Encyclopedia of Genes and Genomes (KEGG) pathway analysis on DEPs. GO terms and KEGG pathways with Benjamini-corrected *p* values < 0.05 and FDR < 0.01 were considered significant.

### PPI network construction and screening of hub proteins

The STRING database (http://string-db.org/) was used to search for the relationship between DEPs and construct the PPI network with a combined score over 0.4, which was considered statistically significant. The PPI networks were visualized by Cytoscape (version 3.9.1) with a hiding of unconnected proteins. The key functional modules of proteins in networks were analyzed by the molecular complex detection technology (MCODE) plug-in in Cytoscape with a degree cutoff = 2, node score cutoff = 0.2, k-score = 2, and max depth = 100. We assessed the top 10 or 15 proteins among the DEPs using seven algorithms, including MCC, MNC, degree, closeness, radiality, stress, and EPC, in the cytoHubba plug-in of Cytoscape. We considered the intersection of common proteins in seven algorithms and proteins in key clusters with the highest scores as hub proteins in networks. Coexpression networks of hub proteins were constructed using the GeneMANIA database.

### Statistical analysis

The differences in data on clinical characteristics, which were presented as the mean or frequency, were tested with a two-tailed independent sample t-test or chi-square test, respectively. The permutation test for one-way ANOVA was used to analyze the differences in NPX between the three groups. A Benjamini‒Hochberg corrected *p-value* < 0.05 indicates a statistically significant difference. Heatmaps of DEPs were generated by pheatmap in the R package. The differences in the relative expression levels of hub proteins or overlapping proteins were tested by permutation test and are displayed in boxplots generated by “ggplot2” in the R package. Correlations between differentially expressed proteins and clinical traits of CMDs were determined using Spearman’s rank correlation coefficients and corresponding *p* values and are presented in a correlation heatmap generated by pheatmap in the R package. Receiver operating characteristic (ROC) curve analysis was performed on overlapping proteins to verify their accuracy, and those with an AUC > 0.7 were deemed useful for disease diagnosis.

## Results

### Baseline characteristics of study participants

The three groups in this study included HY individuals, HE individuals, and elderly individuals with CMDs. Principal component analysis (PCA) based on the samples was first performed for quality control. The results showed that one of the CMDs samples deviated from the group whose IQR was beyond the mean IQR +/- IQR_outlierDef standard deviation. Hence, its data were removed from further analysis (Figure S1). Table 1 shows the baseline characteristics of the subjects. The average age of the HY group was 28.60 (3.53), and that of the HE group and the CMDs group was 80.7 (4.69) and 79.3 (4.67), respectively. In the three groups, there were no statistically significant differences in the plasma indexes that represented the basic metabolic function of the liver and kidney, such as ALT, AST, TBIL, Scr, and BUN. Of note, elderly individuals with CMDs presented higher triglyceride (TG) and FPG levels than HE individuals, while other serum lipids, such as TC, HDL-C, and LDL-C, in the two groups were not significantly different.

**Table 1:** Baseline Characteristic of study participants

	Healthy young	Healthy elderly	Elderly with CMD
Age	28.60 (3.53)	80.7 (4.69) ^a^	79.3 (4.67)
Sex (F/M)	5/5	5/5	6/3
Abdominal obesity	0/10	0/10	10/10 ^b^
CVDs (atherosclerosis, sroke, myocardial infarction, etc.)	0/10	0/10	0/10
ALT (U/L)	21.58 (5.28)	16.56 (4.90)	24.04 (15.55)
AST (U/L)	20.19 (3.04)	22.48 (4.76)	26.39 (14.12)
TBIL (µmol/L)	11.53 (3.47)	12.45 (3.75)	11.98 (3.21)
Scr (µmol/L)	65.47 (13.16)	71.25 (11.58)	71.05 (16.62)
BUN (mmol/L)	5.75 (1.39)	5.50 (0.53)	6.85 (1.88)
TC (mmol/L)	4.72 (0.66)	5.64 (1.10) ^a^	6.00 (1.11)
TG (mmol/L)	1.08 (0.23)	1.21 (0.32) ^a^	2.50 (0.89) ^b^
LDL-C (mmol/L)	3.15 (0.56)	3.38 (0.39)	3.59 (0.83)
HDL-C (mmol/L)	1.25 (0.06)	1.4 (0.34)	1.11 (0.29)
FPG (mmol/L)	4.58 (0.60)	5.61 (0.27)	10.05 (3.03) ^b^

Data are presented as mean (SD) except sex presented as frequency. ALT, glutamic-pyruvic transaminase; AST, glutamic-oxaloacetic transaminase; TBIL, total bilirubin; Scr, serum creatinine; BUN, blood urea nitrogen; TC, total cholesterol; TG, triglyceride; LDL-C, low-density lipoprotein cholesterol; HDL-C, high-density lipoprotein cholesterol; FPG, fasting plasma glucose; ^a^*p* < 0.05 vs. healthy young; ^b^*p* < 0.05 vs. healthy elderly

### Differentially expressed inflammatory proteins between healthy young and healthy elderly individuals

**Fig. 2:**
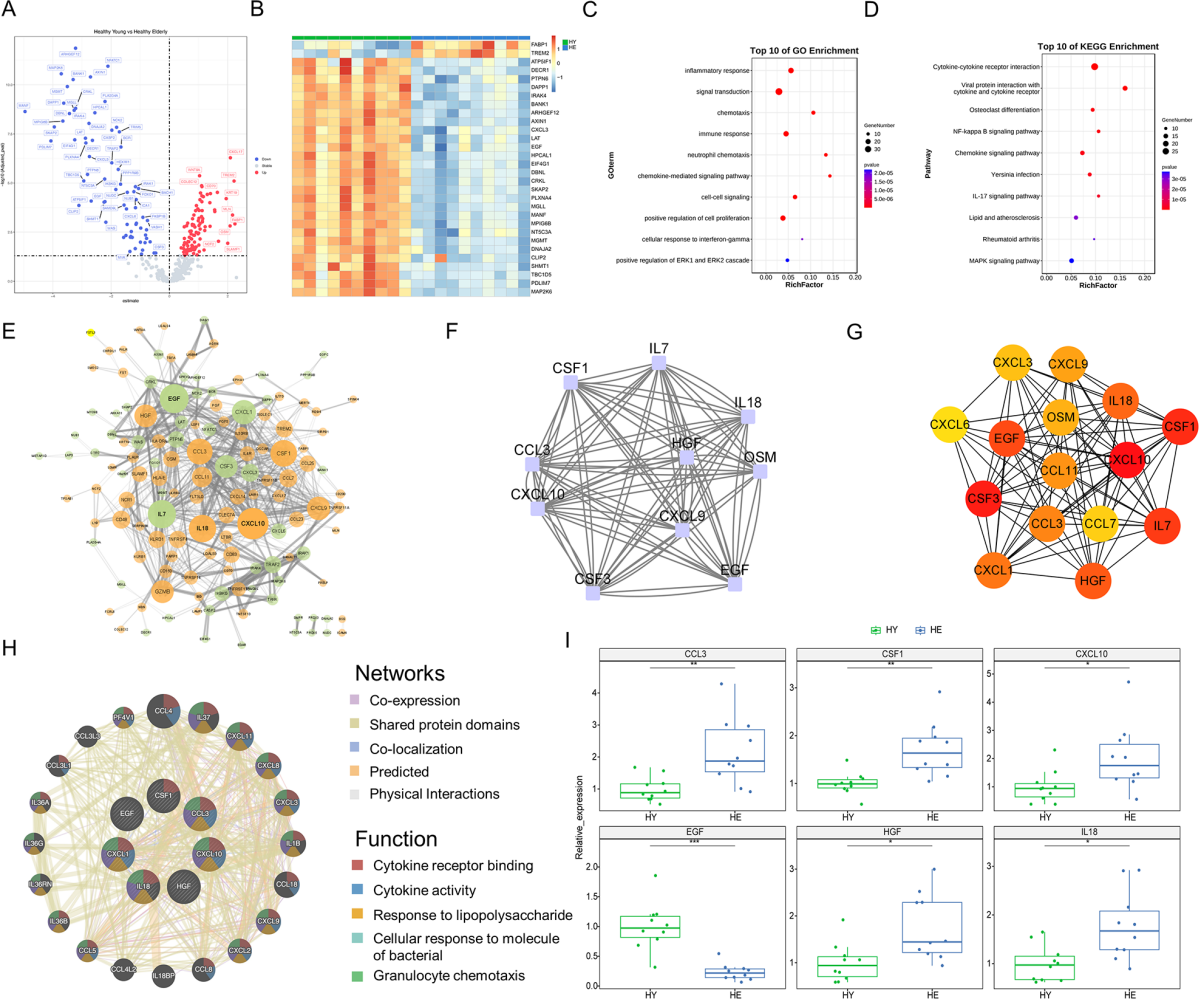
Differentially expressed inflammatory proteins between healthy young and healthy elderly individuals. (**A**) Volcano map of differentially expressed proteins between healthy young and healthy elderly individuals. (**B**) Heatmap shows the top 30 differentially expressed proteins (*p* < 0.05) in healthy young individuals and healthy elderly individuals. (**C**, **D** The enrichment analysis results of GO and KEGG pathways are presented by bubble graphs. An adjusted *p-value* < 0.05 was considered significant. (**E**) PPI network. Node size indicates the number of proteins that interact with it. The green node represents downregulated proteins, and the red node represents upregulated proteins. Edge width indicates the strength of the interaction between the two proteins. (**F**) The node protein clusters with the highest scores were constructed by the MCODE plug-in in Cytoscape. (**G**) The top 15 hub proteins were constructed by cytoHubba. The figure shows the top 15 hub genes constructed by the MCC method. (**H**) The common hub proteins calculated by seven algorithms of plug-in cytoHubba and their coexpressed genes were analyzed via GeneMANIA. (**I**) Relative expression of common hub proteins in DEPs between healthy young individuals and healthy elderly individuals

The flow chart for this research is shown in Fig. 1. We detected 368 inflammatory proteins in the plasma of the three groups by O-link. The comparison of inflammatory proteins between the HY group and HE group indicated significant differences in 161 proteins (adjusted *p* < 0.05) at the plasma level. Of these, 96 inflammatory proteins were upregulated, and 65 inflammatory proteins were downregulated with aging (Fig. 2A). The top 30 DEPs are presented in Fig. 2B. To study the functions of DEPs associated with age, GO biological process and KEGG pathway enrichment analyses were performed. The results showed that DEPs associated with aging were mainly enriched in chemotaxis in GO analysis and NF-κB signaling pathway in KEGG pathway analysis (Fig. 2C and D). To explore the interaction between DEPs, a PPI network with combined scores greater than 0.4 was constructed using the STRING database. The PPI network of DEPs between HY and HE showed that CXCL10, EGF, IL7, IL18, CSF1, CCL3, CXCL1, CXCL9, and CSF3 most closely interacted with other proteins (Fig. 2E). To determine the hub genes in DEPs between HY and HE, functional modules of the PPI network were constructed by the MCODE plug-in in Cytoscape. The cluster with the highest score (9.56) contained 86 edges and 10 nodes, including CXCL10, CCL3, CSF1, CSF3, IL7, IL18, HGF, CXCL9, OSM, and EGF (Fig. 2F). Furthermore, we utilized seven algorithms to screen the top 15 hub proteins. There were 7 proteins in all 7 methods, namely, CXCL10, CSF1, EGF, HGF, IL18, CCL3, and CXCL1 (Table S1 and Fig. 2G). Of these, CXCL10, CSF1, EGF, HGF, IL18, and CCL3 also exist in the functional module. Therefore, these 6 proteins were considered hub proteins of inflammation in aging in this study and were used to construct a coexpression network for analyzing the functions of hub proteins using the GeneMANIA database (Fig. 2H). The relative expression levels of hub proteins are presented in Fig. 2I, which shows that EGF was the most significantly differentially expressed hub protein in aging.

### Differentially expressed inflammatory proteins between healthy elderly individuals and elderly individuals with CMDs

**Fig. 3:**
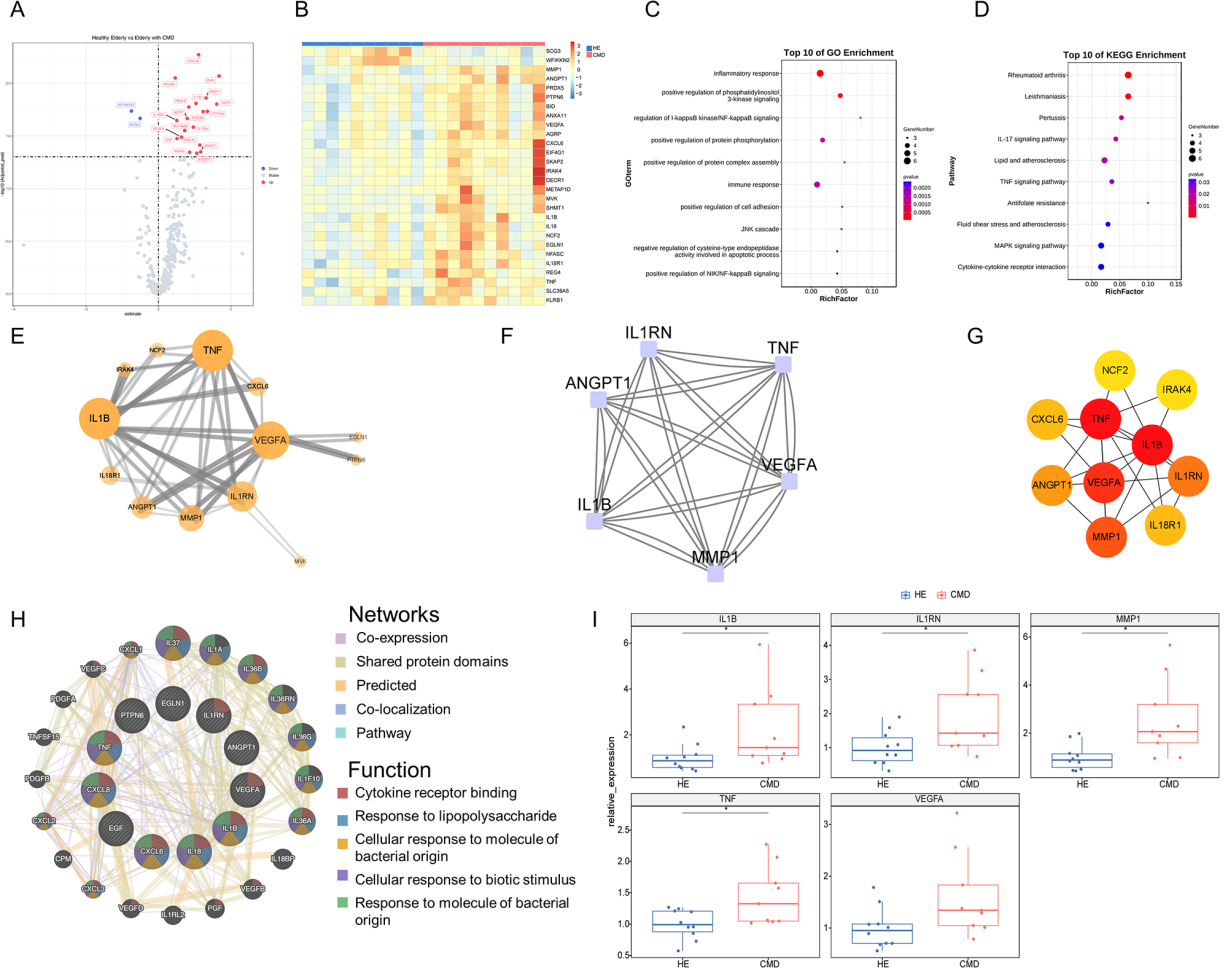
Differentially expressed inflammatory proteins between healthy elderly individuals and elderly individuals with CMDs. (**A**) Volcano map of DEPs between healthy elderly individuals and elderly individuals with CMDs. (**B**) Heatmap shows the differentially expressed proteins (*p* < 0.05) in healthy elderly individuals and elderly individuals with CMDs. (**C**, **D**) The enrichment analysis results of GO and KEGG pathway analyses. An adjusted *p-value* < 0.05 was considered significant. (**E**) PPI network. Node size indicates the number of proteins that interact with it. Edge width indicates the strength of the interaction between the two proteins. (**F**) The node protein clusters with the highest score are constructed by the MCODE plug-in in Cytoscape. (**G**) The top 10 hub proteins were constructed by cytoHubba. The figure shows the top 10 hub genes constructed by the MCC method. (**H**) The common hub proteins calculated by seven algorithms of plug-in cytoHubba and their coexpressed genes were analyzed via GeneMANIA. (**I**) Box plot showing the relative expression of common hub proteins in DEPs between healthy elderly individuals and elderly individuals with CMDs

Compared with HE, CMDs presented significantly different expressions of 28 proteins. Among these, there were 26 upregulated proteins and 2 downregulated proteins (SCG3 and WFIKKN2) (Fig. 3A and B). To study the function of DEPs between HE and CMDs, the results of enrichment analysis indicated that the DEPs were enriched in positive regulation of phosphatidylinositol 3-kinase signaling of GO analysis (Fig. 3C) and IL-17 signaling pathway of KEGG pathway analysis (Fig. 3D). Through protein-protein interaction analysis between DEPs, the network was constructed and consisted of 13 nodes and 54 edges showing that TNF, IL1B, VEGFA, IL1RN, and MMP1 were closely connected with the other proteins (Fig. 3E). Module analysis was used to determine the key cluster in the network of DEPs between HE and CMDs. The functional module consisted of 6 nodes and 28 edges, including TNF, IL1RN, IL1B, MMP1, VEGFA and ANGPT1 (Fig. 3F). In addition, the top 10 hub proteins of the DEPs were analyzed by 7 algorithms, which are shown in Table S2 and Fig. 3G. The common hub proteins include TNF, IL1B, IL1RN, VEGFA, MMP1, IL18R1 and IRAK4. TNF, IL1B, IL1RN, VEGFA, and MMP1 exist in the cluster with the highest score as well. Accordingly, the above 5 proteins were deemed hub proteins in elderly individuals with CMDs, and the co-expression network and functions of these proteins were analyzed as shown in Fig. 3H. Further analysis showed the relative expression of hub proteins, and MMP1 was the most significant hub protein in CMDs (Fig. 3I).

### Common DEPs between normal aging and CMDs

**Fig. 4:**
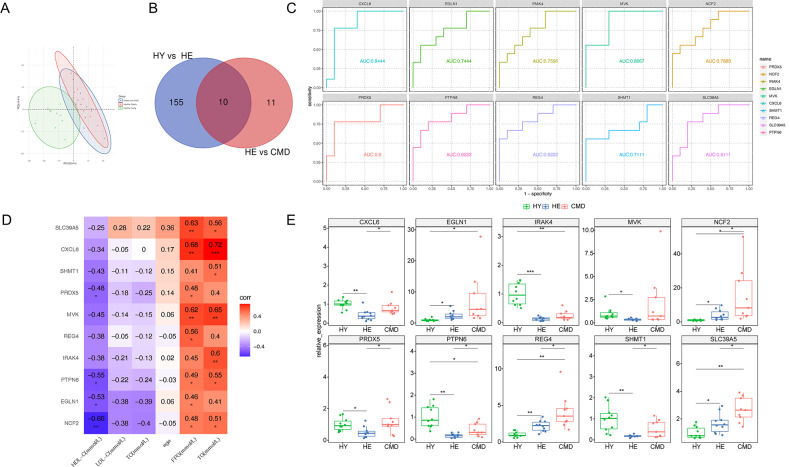
Common DEPs between normal aging and CMDs. (**A**) PCA. The first two primary components (PC1 and PC2) are plotted. (**B**) Venn diagram shows an overlap of differentially expressed proteins between healthy young individuals vs. healthy elderly individuals and healthy elderly individuals vs. elderly individuals with cardio-metabolic disorder. (**C**) ROC curve analysis of overlapping proteins. (**D**) Correlation between differentially expressed proteins and clinical traits in cardio-metabolic disorder. Each cell contains a correlation coefficient. *p < 0.05, **p < 0.01, ***p < 0.001. (**E**) Box plot showing the relative expression of overlapping proteins

Based on the expression of inflammatory proteins, principal component analysis (PCA) indicated that there was the largest difference between HY and HE, but the separation between HE and CMDs was less pronounced (Fig. 4A). The Venn diagram showed that there were 10 overlapping proteins: PRDX5, NCF2, IRAK4, EGLN1, MVK, CXCL6, SHMT1, REG4, SLC39A5 and PTPN6 (Fig. 4B). The related functions of these DEPs are shown in Table S3. To verify the accuracy of overlapping proteins for the diagnosis of CMDs in aging, we performed ROC analysis. The results showed that these proteins had good diagnostic value for CMDs with AUCs > 0.7, which were 0.8, 0.7889, 0.7556, 0.7444, 0.8667, 0.8444, 0.7111, 0.8222, 0.8111 and 0.8222 for PRDX5, NCF2, IRAK4, EGLN1, MVK, CXCL6, SHMT1, REG4, SLC39A5 and PTPN6, respectively (Fig. 4C). We also determined the correlation between these DEPs and clinical traits and found that all overlapping proteins correlated with the level of HDL-C, TG, or FPG in plasma (Fig. 4D). These results suggested that the overlapping proteins have good predictive value for CMDs in aging. We further explored the relative expression of overlapping proteins and found that EGLN1, NCF2, REG4, and SLC39A2 significantly increased in both normal aging and aging with CMDs (Fig. 4E).

## Discussion

Our study identified 368 inflammatory proteins associated with aging and cardio-metabolism in healthy young individuals, healthy elderly individuals, and elderly individuals with CMDs. A total of 161 out of 368 inflammatory proteins may be regulated by aging and be enriched in chemotaxis. Twenty-eight of 368 inflammatory proteins may be associated with CMDs and be enriched in I-κB kinase/NF-κB signaling. There were 10 common inflammatory proteins possibly related to both aging and CMDs, namely, SHMT1, MVK, EGLN1, SLC39A5, NCF2, CXCL6, IRAK4, REG4, PTPN6, and PRDX5. This study may presented a novel inflammatory protein profile in plasma associated with aging and CMDs and revealed their common inflammatory molecule phenotypes.

While most of the identified inflammatory biomarkers would seem to represent novel findings related to aging, some of them have been reported in several proteomics studies of aging. A previous study identified 217 proteins in plasma that are significantly associated with age by measuring 1301 proteins in 240 healthy subjects aged 22–94 years [21]. Among them, 14 proteins were also present among the 165 inflammatory biomarkers identified in this study, namely, CXCL10, CCL11, CHRDL1, CCL23, EGF, SPON1, FSTL3, REG4, CCL3, CXCL9, LGALS9, PLAUR, CCL7 and EPHA1. Furthermore, many proteomic studies of age-related biomarkers performed in different matrices using multiple platforms were reviewed. In the literature, they reported 232 age-associated biomarkers that had a consistent direction across at least two different studies and were associated with age in at least one other nonplasma matrix regardless of the direction [22]. Notably, 6 biomarkers were also identified in this study, including EGF, SPON1, LGALS4, CRKL, PLAUR, and BSG. However, none of the biomarkers in our study were present in senescence-associated secretory phenotypes (SASPs) previously identified in several types of senescent cells induced by senescence-inducing stimuli in vitro [23, 24]. The possible reason is that their study subjects and experimental context are largely different. Our study suggested that CXCL10, CSF1, EGF, HGF, IL18, and CCL3 were hub proteins related to age among 161 inflammatory proteins, and EGF seems to be the most significant age-associated protein. Although few studies have proven the decline in EGF in normal aging, the level of EGF decreases in the early stage of Parkinson’s disease, which is related to the nonmotor symptoms of PD patients [25]. The concentration of EGF in plasma increased in patients with mild cognitive impairment and Alzheimer’s disease. Interestingly, EGF conferred a protective effect on cognitive and cerebrovascular dysfunction in an AD-Tg mouse model that incorporates cerebrovascular-relevant AD risk factors [26]. Therefore, it is worth exploring the relationship between changes in plasma EGF and aging and age-related diseases in a large-scale population.

CVDs are prevalent in the aging population, and cardiometabolic disorders are a major atherogenic risk factor that subsequently elicits cardiovascular diseases [27]. Thus, our study suggests that there may be a link between 28 inflammatory proteins and CMDs. WFIKKN2 is the only protein that was reported in a previous plasma proteomic study that presented 53 proteins associated with metabolic syndrome defined by the simultaneous clustering of cardio-metabolic risk factors, although it was determined to have no causal effects on metabolic syndrome [28]. Nonetheless, it was claimed that there was a bidirectional causal relationship of WFIKKN2 with BMI, which is an important risk factor for cardiovascular diseases [29]. In addition, some main circulating biomarkers in metabolic syndrome were not present in 22 inflammatory proteins in our study, such as IL6, which is linked to low HDL-C and high TGs, impairment of glucose metabolism, vascular dysfunction, and atherosclerosis [4].

In addition to the above proteins, we may present several newly identified potential inflammatory proteins associated with CMDs in plasma. Among them, TNF, IL1B, IL1RN, VEGFA, and MMP1 are hub proteins selected by many algorithms, and MMP1 is the most significant. Circulating MMP1 levels have been reported to correlate with coronary artery disease burden (number of diseased coronary arteries ≥ 50% stenosis) and promote atherosclerosis progression by atypically activating PAR1 signaling and contributing to the amplification of TNF-α signaling in endothelial cells. Clinical trials and experiments in mice have demonstrated that MMP1 blockade or deficiency results in a reduction in total aortic plaque burden and the number of macrophages in plaques [30–32]. MMP1 levels also had a strong positive correlation with FPG through correlation analysis, which indicates that MMP1 is a vital molecule in CVDs.

The overlap between differentially expressed inflammatory proteins of aging and CMDs may reveal the common molecular alterations between aging and CMDs. Among these proteins, EGLN1, NCF2, REG4, and SLC39A2 were prominently increased both in normal aging and aging with CMDs. Notably, NCF2 was the most differentially expressed protein in these ten common proteins. It is one of a group of proteins that forms an enzyme complex called NADPH oxidase, which plays an essential role in the immune system. A previous study determined that its expression was elevated in the skeletal muscle of obese individuals compared with lean subjects [33]. However, NCF2 has rarely been reported in CVDs and aging or aging-related diseases. This suggests that NCF2, as an important regulator in the immune system, has great study potential for CVDs and aging or aging-related diseases.

EGLN1 and SLC39A5 have been reported to contribute to aging-related diseases or metabolic dysfunction. Specifically, EGLN1 targets HIF subunits for proteasomal destruction under normal oxygen concentrations. Inhibition of it improves glucose and lipid metabolism and protects against obesity and metabolic dysfunction^42^. Furthermore, EGLN1 blockade was determined to have a beneficial impact on atherosclerosis by reducing approximately 50% of atherosclerotic plaque areas and macrophage numbers in white adipose tissue, increasing autoantibodies against oxidized LDL [34, 35]. In a study of age-related neurodegeneration, EGLN1-HIFa signaling contributed to mitochondrial stress-induced neurotoxicity by ATP13A2 regulation in Parkinson’s disease (PD) [36]. EGLN1 inhibition was verified to maintain cellular iron hemostasis and neuronal viability in a PD mouse model and cultured human DAergic cells [37]. Conversely, SLC39A5 is indispensable for glucose metabolism, and it was reported that SLC39A5 is significantly downregulated in diabetic mice and that β-cell-specific Slc39a5 knockout mice have impaired insulin secretion [38].

It is necessary to acknowledge the limitations of this study. Firstly, the sample size of our study is small. The primary focus of this study was to investigate potential inflammatory biomarkers associated with aging and metabolic abnormalities, as well as the progression to cardiovascular diseases from the pathogenesis of metabolic disorders. However, the majority of patients presenting at the hospital exhibit cardiovascular conditions, with a minimal portion of elderly patients displaying metabolic abnormalities without a confirmed diagnosis of cardiovascular diseases. The findings should be further investigated in trials with a larger patient cohort. Secondly, the clinical pairs were not strictly matched across the three distinct groups, our results did not completely exclude the impact of covariates on the expression of inflammatory proteins in subjects.

## Conclusion

This study may reveal the alteration characteristics of serous inflammatory proteins in aging or CMDs and common molecular changes between aging and CMDs. It showed a signature pattern that could not only provide information regarding the future risk of CMDs and metabolic function decline but also clue on the mechanism by which aging contributes to CMDs. Moreover, to verify the predictive value of common proteins, further studies are needed to follow up and explore the correlation between protein levels and the occurrence of cardiovascular and cerebrovascular diseases in elderly individuals and those with CMDs.

### Electronic supplementary material

Below is the link to the electronic supplementary material.

Supplementary Material 1

## Data Availability

No datasets were generated or analysed during the current study.

## Abbreviations

ALT: glutamic-pyruvic transaminase
AST: glutamic-oxaloacetic transaminase
BUN: blood urea nitrogen
CMDs: cardiometabolic disorders
CVDs: cardiovascular diseases
DEPs: differentially expressed proteins
FPG: fasting plasma glucose
FPG: fasting plasma glucose
GO: Gene Ontology
HE: healthy elderly
HY: healthy young
HDL-C: high-density lipoprotein cholesterol
KEGG: Kyoto Encyclopedia of Genes and Genomes
LDL-C: low-Density lipoprotein cholesterol
MCODE: molecular complex detection technology
PD: Parkinson’s disease
PCA: Principal component analysis
PPI: protein‒protein interaction (PPI)
PEA: Proximity Extension Assay technology
ROC: receiver operating characteristic
Scr: serum creatinine
SASPs: senescence-associated secretory phenotypes
TBIL: total bilirubin
TC: total cholesterol
TG: triglyceride
