# Supplementary material for: Multiplex proteomics identifies inflammation-related plasma biomarkers for aging and cardio-metabolic disorders
Source: Clin Proteomics. 2024 Apr 22;21:30. doi: 10.1186/s12014-024-09480-x (PMC11036613; doi:10.1186/s12014-024-09480-x)
Supplement: Supplementary file 1 — Supplementary Material 1 [file 12014_2024_9480_MOESM1_ESM.docx]

**SUPPLEMENT**


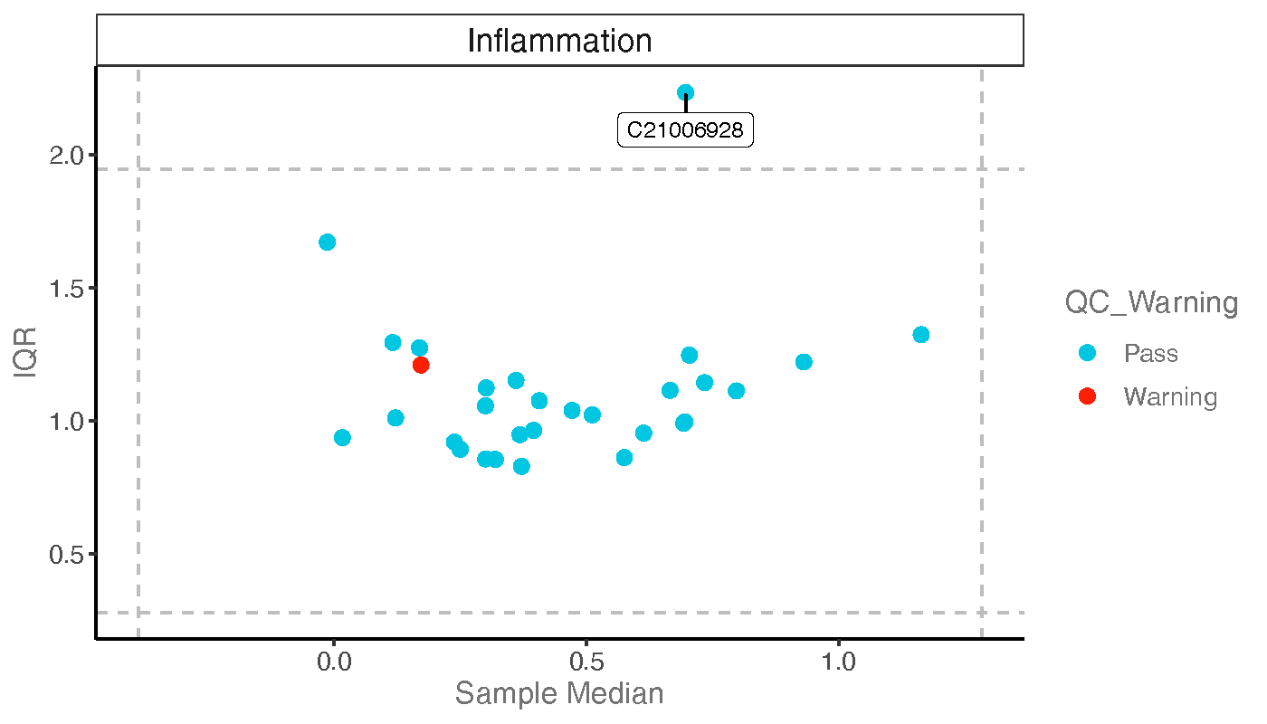


**Figure S1. PCA analysis base on sample**

Horizontal dashed lines indicate +/-IQR_outlierDef standard deviations from the mean IQR. Vertical dashed lines indicate +/-median_outlierDef standard deviations from the mean sample median)

**Table S1. The top 15 hub proteins of DEPs in aging rank in cytoHubba**

| **MCC** | **MNC** | **Degree** | **EPC** | **Closeness** | **Radiality** | **Stress** |
| --- | --- | --- | --- | --- | --- | --- |
| CXCL10 | CXCL10 | CXCL10 | CXCL10 | CXCL10 | CXCL10 | EGF |
| CSF3 | IL7 | EGF | IL18 | EGF | EGF | PLAUR |
| CSF1 | IL18 | IL7 | IL7 | IL18 | IL18 | HGF |
| IL7 | CSF1 | IL18 | CSF1 | IL7 | IL7 | CTRC |
| EGF | CCL3 | CSF1 | CCL3 | CCL3 | CCL3 | IL18 |
| HGF | EGF | CCL3 | CXCL1 | CSF1 | CSF1 | GZMB |
| IL18 | CXCL9 | CXCL1 | CXCL9 | CXCL9 | CXCL9 | CXCL10 |
| CXCL1 | CXCL1 | CXCL9 | EGF | CXCL1 | CXCL1 | CLEC7A |
| CCL3 | CSF3 | GZMB | CSF3 | HGF | HGF | CCL3 |
| CCL11 | HGF | HGF | CCL11 | GZMB | GZMB | FST |
| CXCL9 | GZMB | CSF3 | GZMB | CSF3 | CSF3 | CXCL1 |
| OSM | CCL11 | CCL11 | CXCL3 | CCL11 | CCL11 | PARP1 |
| CXCL3 | CXCL3 | CD48 | HGF | CCL7 | CCL7 | LAP3 |
| CCL7 | CCL7 | CXCL3 | CCL7 | KLRD1 | KLRD1 | CSF1 |
| CXCL6 | TRAF2 | CCL7 | CCL25 | OSM | OSM | CD48 |

**Table S2. The top 10 hub proteins of DEPs in CMD rank in cytoHubba**

| **MCC** | **MNC** | **Degree** | **EPC** | **Closeness** | **Radiality** | **Stress** |
| --- | --- | --- | --- | --- | --- | --- |
| TNF | TNF | TNF | TNF | TNF | TNF | VEGFA |
| IL1B | IL1B | IL1B | IL1B | IL1B | IL1B | TNF |
| VEGFA | VEGFA | VEGFA | VEGFA | VEGFA | VEGFA | IL1B |
| MMP1 | MMP1 | IL1RN | IL1RN | IL1RN | IL1RN | IL1RN |
| IL1RN | IL1RN | MMP1 | MMP1 | MMP1 | MMP1 | MMP1 |
| ANGPT1 | ANGPT1 | ANGPT1 | ANGPT1 | ANGPT1 | ANGPT1 | EGLN1 |
| IL18R1 | IL18R1 | IL18R1 | CXCL6 | CXCL6 | CXCL6 | PTPN6 |
| CXCL6 | CXCL6 | CXCL6 | IL18R1 | IL18R1 | IL18R1 | IL18R1 |
| IRAK4 | IRAK4 | IRAK4 | NCF2 | IRAK4 | IRAK4 | IRAK4 |
| NCF2 | NCF2 | NCF2 | IRAK4 | NCF2 | NCF2 | MVK |

**Table S3. The function of overlapping proteins.**

|  | **Proteins** | **Function** |
| --- | --- | --- |
| Metabolism | SHMT1 | Serine Hydroxymethyltransferase 1, a pyridoxal phosphate-containing enzyme that can provide one-carbon units for synthesis of methionine, thymidylate, and purines in the cytoplasm |
|  | MVK | Mevalonate Kinase, is an enzyme in the cholesterol synthesis pathway. This pathway is responsible for the synthesis of sterol products and nonsterol isoprenoids. |
|  | EGLN1 | Egl-9 Family Hypoxia Inducible Factor 1, catalyzes the post-translational formation of 4-hydroxyproline HIF-a proteins. Prolyl hydroxylation of HIF is a key regulatory event that targets HIF subunits for proteasomal destruction under normal oxygen concentration |
|  | SLC39A5 | Solute Carrier Family 39 Member 5, belongs to the ZIP family of zinc transporters that transport zinc into cells from outside, and play a crucial role in controlling intracellular zinc levels. |
| Inflammation | NCF2 | Neutrophil cytosolic factor 2, is one part of a group of proteins that forms an enzyme complex called NADPH oxidase, which plays an essential role in the immune system |
|  | CXCL6 | C-X-C Motif Chemokine Ligand 6, is a chemotactic for neutrophil granulocytes and has antibacterial action against gram-negative and gram-positive bacteria. |
|  | IRAK4 | Interleukin 1 Receptor Associated Kinase 4, IRAK-4-dependent Toll and IL-1R pathway leads to the synthesis of inflammatory cytokines, after TLR7, TLR8, and TLR9 stimulation |
| Proliferation and regeneration | REG4 | Regenerating Family Member 4. Within the REG family, REG4, located in cytoplasm, is involved in cell proliferation and regeneration. Enables heparin binding activity and mannan binding activity. |
| Signaling transduction | PTPN6 | Protein Tyrosine Phosphatase Non-Receptor Type 6, is expressed primarily in hematopoietic cells, and functions as an important regulator of multiple signaling pathways in hematopoietic cells |
| Oxidative stress | PRDX5 | Peroxiredoxin 5, is a member of the peroxiredoxin family of antioxidant enzymes which interacts with peroxisome receptor 1 and plays an antioxidant protective role in different tissues under normal conditions and during inflammatory processes. |
